# Treatment of Melancholia

**Published:** 1885-12

**Authors:** 


### Treatment of Melancholia.

Dr. DeFoe writes as follows: "The family doctor only knows how widespread melancholia is in our country. The many household cares develop this disease in nervous women, who show its first symptoms in fretfulness and worry. I have sought for a remedy for years for this malady, and have at last found it in the triple valerianates, which work like a charm:

| | |
|---|---|
| Zinci Valerianat, - - - - - - | 20 grains. |
| Quiniæ Valerian, - - - - - - | 20 " |
| Ferri Valerian, - - - - - - | 20 " |

[M. ft. pil. No. 20. Sig.: One, three times a day.]

"The drugs must be absolutely pure. The old reliable house of W. H. Schieffelin & Co., of New York, have added the above pills (soluble) to their list, and I have tried them in many cases, and I find them a specific for the worry of nervous women, melancholia and incipient insanity.

"Please try them and report. Your success will be sure."—*Medical Brief.*

